# Development of Natural Product-Conjugated Metal Complexes as Cancer Therapies

**DOI:** 10.3390/ijms20020341

**Published:** 2019-01-15

**Authors:** Dik-Lung Ma, Chun Wu, Sha-Sha Cheng, Fu-Wa Lee, Quan-Bin Han, Chung-Hang Leung

**Affiliations:** 1 Department of Chemistry, Hong Kong Baptist University, Kowloon, Hong Kong 999077, China; ccwuchem@gmail.com; 2 State Key Laboratory of Quality Research in Chinese Medicine, Institute of Chinese Medical Sciences, University of Macau, Taipa, Macao 999078, China; yb77535@connect.umac.mo; 3 College of International Education, School of Continuing Education, Hong Kong Baptist University, Shek Mun, Hong Kong 999077, China; fuwalee@hkbu.edu.hk; 4 School of Chinese Medicine, Hong Kong Baptist University, Kowloon, Hong Kong 999077, China; simonhan@hkbu.edu.hk

**Keywords:** cancer therapy, transition metal complex, natural product, cytotoxicity

## Abstract

Platinum-based drugs have revolutionized cancer care, but are unfortunately associated with various adverse effects. Meanwhile, natural product scaffolds exhibit multifarious bioactivities and serve as an attractive resource for cancer therapy development. Thus, the conjugation of natural product scaffolds to metal complexes becomes an attractive strategy to reduce the severe side effects arising from the use of metal bearing drugs. This review aims to highlight the recent examples of natural product-conjugated metal complexes as cancer therapies with enhanced selectivity and efficacy. We discuss the mechanisms and features of different conjugate complexes and present an outlook and perspective for the future of this field.

## 1. Introduction

**Figure 1:**
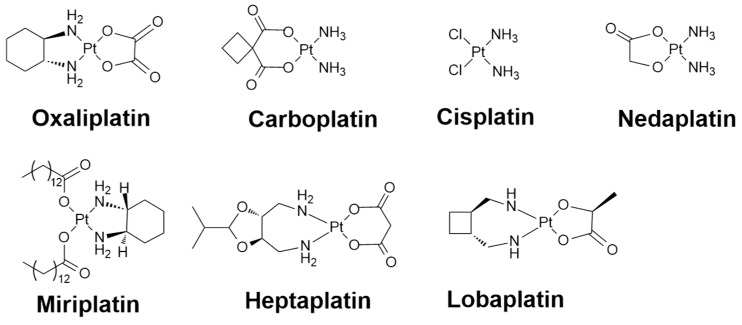
Chemical structure of approved platinum(II) and platinum(IV)based drugs.

Since the serendipitous discovery of cisplatin, platinum-based drugs have emerged and have become one of the most widely-used class of chemotherapeutic drugs against various human tumors, such as testicular cancer, ovarian cancer, lung, head and neck, and advanced bladder cancer [1]. Three platinum(II)-based drugs (oxaliplatin, carboplatin, and cisplatin) are officially approved in the United States, while regionally approved platinum(II) and platinum(IV) drugs are also available in Japan (nedaplatin and miriplatin), Korea (heptaplatin), and China (lobaplatin) (Figure 1) [2,3]. Platinum-based chemotherapies mainly function by entering the target cells under the assistance of copper transporters [4]. Upon entering a cell, aquation of the metal complexes firstly takes place as a result of a low osmotic pressure environment, and the complexes may undergo further functional group hydrolysis for activity activation, particularly in the form of losing labile moiety such as carboxylate and chloride. The metal-based molecules become highly reactive after activation and readily display a positive interaction with the intracellular targeting sites notably embedded in the proteins/peptides or nuclear DNA, especially for redox active sulfur residues from methionine or cysteine [5,6]. Despite the clinical success of platinum-based drugs, their use remains limited by their systemic toxicity, which results in bone marrow suppression, hair loss, vomiting, nausea, and so on [7,8].

## 2. Side Effects of Platinum-Based Cancer Therapies

Platinum-based cancer therapies possess inevitable side effects owing to their limited selectivity for cancer cells or tissues over the surrounding healthy ones. The side effects might be induced by the same high nutrition requirements of cancerous cells versus other fast-growing healthy cells, such as cells of the mucous membranes, bone marrow, and hair follicles [9,10]. Around 40 specific side effects have been documented from the use of platinum-based drugs, which can be classified into the following sub-types: nephrotoxicity, ototoxicity, gastrointestinal toxicity, hepatotoxicity, hematological toxicity, cardiotoxicity, and neurotoxicity [11]. The symptoms generated from the severe side effects mainly include asthenia, anorexia, cachexia, alopecia, pain, stomatitis, mucositis, diarrhea, vomiting, nausea, cytopenias, anaphylaxis, and so on. Hence, patients taking platinum-based chemotherapeutics require additional medical interference and monitoring in order to achieve normal physiological maintenance of the body [12]. Additionally, ancillary drugs are commonly co-prescribed with platinum-centered cancer therapies to minimize unwanted side effects, which can include antioxidants, antibody cytokine blockers, monoclonal, magnesium supplements, saline hyperhydration, propafenone, mannitol, myeloid growth factors, antibiotics, and antiemetics [13,14].

Another important issue for controlling the side effects from platinum-based therapies is to avoid a superfluous aquation reaction, which typically occurs during the drug preparation and administration process. The use of cisplatin is regarded as a pattern metal-based cancer therapy, and cisplatin is commonly formulated in a stabilizer solution containing 0.9% sodium chloride, to ease the problem of losing the chloride ligand from the cisplatin scaffold. However, the sodium chloride solution used in the formulation would lead to the premature structure conversion and activation of parent drugs, like carboplatin or oxaliplatin, into more reactive but less soluble cisplatin or PtCl_2_(R,R-dach) (dach = diaminocyclohexane), respectively. Therefore, a 5% glucose solution, instead of a 0.9% sodium chloride solution, is normally adopted in the carboplatin and oxaliplatin formulation [9,15]. Moreover, it has been well documented that the toxicity of platinum-based cancer therapy is highly related to its level of binding reactivity towards functional target sites, which is determined mostly by the stability of the binding/leaving moieties on the parent drugs. The parent metal-based scaffolds bearing more labile moieties tend to be more active and generate more undesirable side effects at equivalent doses to other platinum drugs [16,17]. For instance, introducing a bis-carboxylate ligand into cisplatin to replace the already conjugated chloride ligands can reduce the aquation reactivity. As a result, a reduction of toxicity is demonstrated [16,18,19].

## 3. Strategies for Reducing the Toxicity of Metal-Based Cancer Therapies

Targeted cancer therapy is a challenging strategy that uses drugs or other substances to more accurately identify and attack cancer cells. One of the approaches is directing the therapeutic molecules selectively to the targeted cancer cells or tissues, and thus promoting the curing efficacy and minimizing the unwanted side effects simultaneously [20,21]. Therefore, the development of efficiently targeted drugs with minimal side effects and toxicity is important for anticancer therapies [20].

Cancer cells/tissues always possess similar characteristics to the homologous generated healthy cells/tissues, rendering selective treatment challenging. The bearing ligands on a metal-centered scaffold play a significant role in tuning the corresponding efficiency and toxicity properties of the anticancer therapy. The modification of the ligands benefits the regulation of the associated substitutional hydrophilicity/hydrophobicity, inertness/oxidation reactivity, and systematic/target biocompatibility according to specific treatment requirements and application conditions [22,23,24]. A hydrolysis process often takes place for the activation of premature metal therapies to allow for further binding to target the intercellular biomolecules [25,26]. Therefore, a high affinity and specificity to the reception targeting sites are always of primary consideration for ligand modification in metal-centered therapy development. Moreover, metal centers might experience a redox reaction for activation [27]. For example, platinum(IV) conjugated prodrugs can undergo reduction and generate platinum(II) complexes after delivering to intra-/inter-cellular target sites. As the platinum(IV)-based complexes are kinetically inert, they will not be as likely to cause side reactions until they enter into the cellular environment. Upon entry into the cell, they undergo reduction to generate the active platinum(II) species that is capable of forming platinum(II)-DNA adducts. The redox mechanism is thought to involve the initial binding of the platinum(IV) complex to the N7 site of the guanosine (G) moiety. Subsequently, the 5′-phosphate or 5′-hydroxyl group attacks the C8 site of the G moiety, resulting in a two-electron transfer process that produces cyclic (5′-*O*-C8)-G and a platinum(II) complex. The platinum(II)–DNA adducts induce the distortion of DNA structures and other biological dysfunctions, leading to cancer cell death [28]. The further modification of auxiliary ligands tunes the hydrophobicity and stability of platinum(IV) conjugated drugs to maintain intact structures before arrival at the cancer cells, with only selective redox reaction on target sites, thus reducing the undesirable toxicity to nearby healthy cells/tissues [29].

Moreover, the replacement of alternative metal centers, such as ruthenium(II), gold(III), palladium(II), iridium(III), rhodium(III), iron(III), osmium(IV), cobalt(II), and tin(II) [30,31,32,33,34], offers promising options to ease drug resistance to cisplatin, and to achieve diverse anticancer activities with reduced sides effect [35,36,37,38]. Ruthenium(II)-centered complexes are of particular interest among these metal centers in virtue of their moderate side effects with their reduced toxicity, excellent selectivity over healthy cells/tissues, and high efficiency of anticancer activity [39], along with their diverse functional mechanisms through different coordination geometries, coordination types, and oxidation states [13,40]. As octahedral ruthenium(II) complexes with three bidentate (usually polypyridyl) ligands are typically propeller-shaped, they possess two enantiomeric configurations that can differ in their biological activity and selectivity against target chiral biomolecules, such as DNA. Generally, the “left” configuration of the ruthenium(II) complexes is more able to bind to the minor groove of the DNA, while the “right” configuration is more likely to intercalate into the DNA molecule [41,42]. Moreover, ruthenium(II) complexes have also been developed that target the major groove of DNA, thereby avoiding the most common mechanisms of drug resistance from platinum-centered cancer therapies [43].

## 4. Natural Product-Conjugated Metal Complex Cancer Therapies

Natural product scaffolds exhibit multifarious bioactivities and serve as an attractive resource for cancer therapy development. The conjugation of natural product scaffolds to metal complexes serves as an attractive strategy to reduce the severe side effects of metal bearing drugs [44,45,46]. In the following sections, representative examples of the conjugation of natural product molecules to metal complexes will be illustrated (Table 1). Natural products possess inherent benefits as medicinal scaffolds, including an abundant structural diversity, intrinsic bioactivity, and excellent biocompatibility. The attachment of natural product moieties onto metal-centered complexes can confer selectivity for intercellular/intracellular target sites and as well as overcome typical mechanisms of drug resistance exhibited by cancer cells against platinum-based drugs. Moreover, the fine-tuning of either the metal centers or the conjugated natural products allows for the optimization of pharmacological characteristics, including enhanced biological potency and reduced side effects, depending on the mechanism of action of the natural product-conjugated metal complex [39,47,48].

### 4.1. Small Molecules

**Figure 2:**
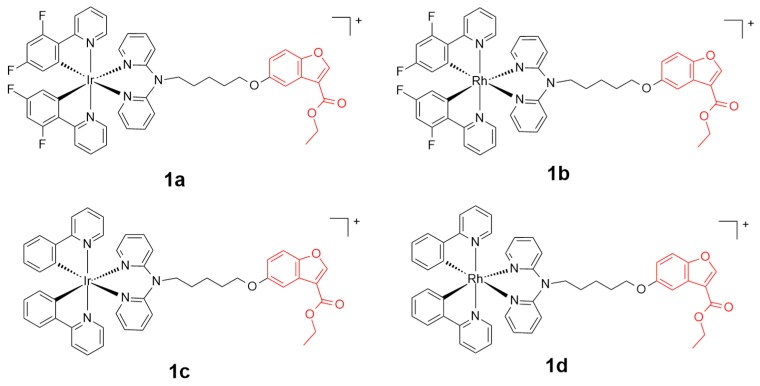
Chemical structure of complexes **1a**, **1b**, **1c**, and **1d**. The benzofuran motif is highlighted in red. Reprinted figure with permission from Copyright (2017) Elsevier B.V.

Benzofuran and its derivatives widely exist in nature and display significant inhibition activities upon comprehensive interaction towards cancer related biomarkers such as β-amyloid, mPTPB (protein tyrosine phosphatase B), mTOR (mammalian target of rapamycin), and PI3 (phosphatidylinositide 3) kinase [49,50]. A series of benzofuran-conjugated rhodium(III) or iridium(III)-centered metal scaffolds **1a**–**1d** (Figure 2) have been reported as anti-prostate cancer agents by Ma and coworkers [51]. Complex **1a** exhibited the best inhibition efficiency among the four scaffolds towards either the TNF-α-induced NF-κB pathway (TNF = tumor necrosis factor; NF-κB = nuclear factor kappa light chain enhancer of activated B cells) or IL-6-induced STAT3 pathway (IL-6 = Interleukin 6; STAT3 = Signal transducer and activator of transcription 3) in DU145 prostate cancer cells. Complex **1a** regulated the protein expression and permutation by the inhibition of the cytoplasm translocation of NF-κB and STAT3 to the nucleus. Additionally, complex **1a** displayed a selective toxicity against DU145 cells and a suppression activity against tumors in a prostate cancer xenograft mouse model. Complex **1a**, with the half maximal inhibitory concentration (IC_50_) at ca. 4.34 µM, showed a higher toxicity against DU145 cancer cell lines than the reference cisplatin and doxorubicin (IC_50_ > 30 µM), probably by disrupting the plasma membrane integrity. Importantly, complex **1a** exhibited a selective inhibition activity for cancer cells over healthy cells, with a relatively lower cytotoxicity against the healthy cells, including HEK293 (IC_50_ = ca. 32.34 µM) and LO2 cells (IC_50_ = ca. 29.21 µM).

**Figure 3:**
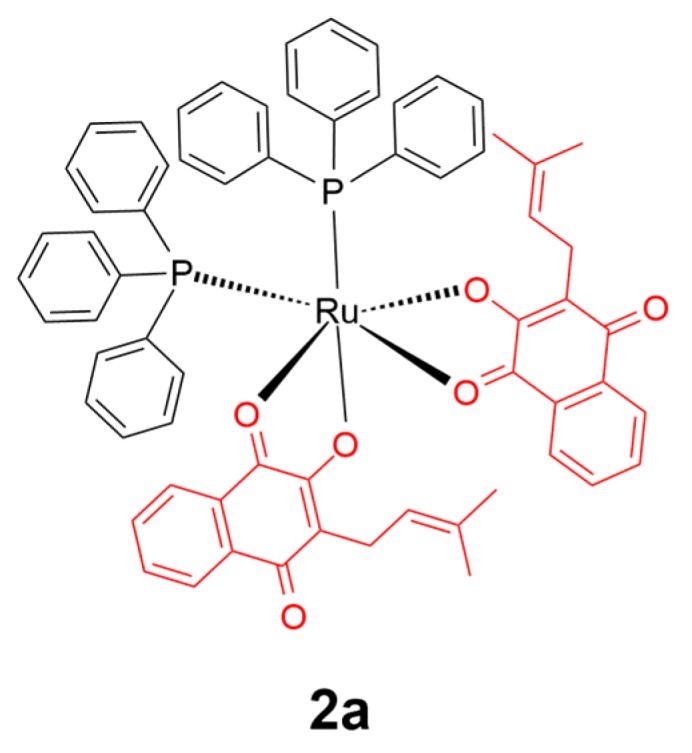
Chemical structure of complex **2a**. The lapachol moiety is highlighted in red. Reprinted figure with permission from Copyright (2017) Elsevier Ltd.

Lapachol is a naphthoquinone natural product mainly derived from Bignoniaceae plants with diverse biological activities including anticancer, antimicrobial, antiparasitic, antifungal, and antiviral activities [46,52]. In 2017, a lapachol conjugated ruthenium(II) (**2a**) scaffold was reported (Figure 3) [53]. Fluorescence measurements suggested that the cis configuration of complex **2a** showed stronger binding upon both bovine serum albumin (BSA) and human serum albumin (HSA) proteins compared to the trans configuration one. However, cytotoxicity assays against healthy lung cells (V79) and breast/lung cancer cells (MDA-MB-231/A549) indicated that trans-**2a** was more active and selective towards cancer cells over healthy cells than both the cis isomer and the reference drug cisplatin, which was attributed to the weaker affinity of trans-**2a** to engage in non-selective interactions with non-target proteins such as albumin.

**Figure 4:**
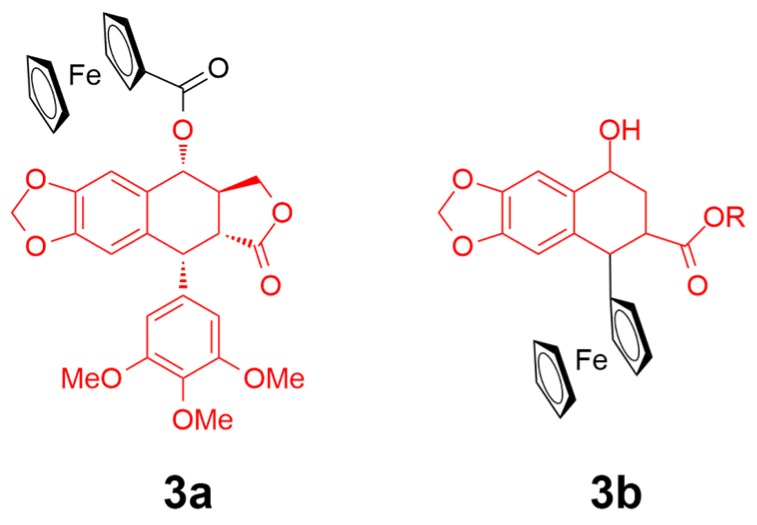
Chemical structure of complexes **3a** and **3b**. The podophyllotoxin moiety and analogue are highlighted in red. Reprinted figure with permission from Copyright (2017) Elsevier B.V.

Podophyllotoxin (PPT) is a root-derived natural product from Podophyllum peltatum that exhibits a tubulin/antimitotic targeting activity, resulting in the interference of cell division or even cell death [54]. PPT possesses a high cytotoxicity and demonstrates severe side effects, such as vomiting, diarrhea, and nausea, and a poor selectivity over targeting cells [55]. Recently, conjugated ferrocenyl–podophyllotoxin analogues have been reported as breast cancer inhibitors (Figure 4) [56]. Podophyllotoxin alone shows a high activity against cancer cells MDA-MB-231 and MCF-7 (IC_50_ = 0.01 µM). Upon the conjugation of PPT to ferrocenyl complexes, the cytotoxicity of **3a** was decreased with an IC_50_ value of 0.43 and 0.93 µM against the MDA-MB-231 and MCF-7 cells, respectively. However, the PPT analogue-conjugated **3b** was found to be much less potent than **3a**. On the basis of the reversible redox behavior of the ferrocenyl moiety, which might affect the cellular oxidative environment and facilitate the generation of reactive oxygen species (ROS), the authors suggested that the metal-complex–PPT conjugate could show superior selectivity for cancer cells over healthy ones.

**Figure 5:**
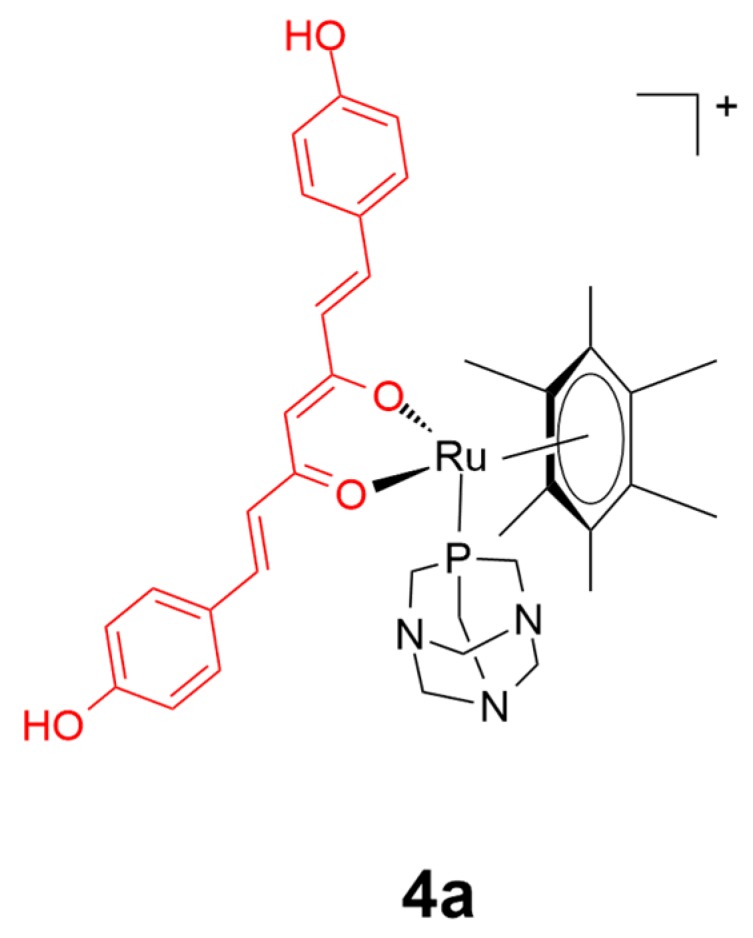
Chemical structure of complex **4a**. The curcumin moiety is highlighted in red. Reprinted figure with permission from Copyright (2014) American Chemical Society.

Curcumin is the principal active ingredient from the herbal plant *Curcuma longa*. Curcumin has been extensively studied as a pharmacological agent because of its broad range of biological activities, including its anticancer, antioxidant, and anti-inflammatory effects [57,58]. However, as curcumin itself is both unstable and not readily bioavailable [59,60], researchers have investigated whether the attachment of curcumin to metal scaffolds could allow for the favorable medicinal properties of both curcumin and the metal complex to be maintained, and yet retaining the desirable biocompatibility. The first curcumin-based metal complex was reported in 1987, which was a gold(III) conjugate that displayed moderate anti-arthritic activity [61]. Subsequently, curcumin complexes based on vanadyl(II), nickel(II), cobalt(II), copper(II), ruthenium(II), palladium(II), and zinc(II) have been reported [45,62]. Among these, the ruthenium(II) conjugates have shown the greatest potential as anticancer agents. For instance, a series of novel ruthenium(II) complexes have been developed by Dyson, Pettinari, and coworkers, using the conjugation of 1,3,5-triaza-7-phosphaadamantane (RAPTA) and curcumin as auxiliary ligands [63]. All of the curcumin complexes showed both an improved solubility and high selectivity for the tumor cell lines (A2780 and A2780cisR) over the non-tumorous HEK293 cell line. Particularly, compound **4a** (Figure 5) showed the most promising activity profile over cisplatin, with around a 70-fold higher inhibition activity against the cancer cell lines (IC_50_ < 0.27 μM) over the normal HEK cell line (IC_50_ = 13.0 μM). The replacement of the chloride ligand present in most platinum drugs with the RAPTA ligand was thought to allow the complexes to bypass drug resistance mechanisms for cisplatin. Moreover, the more rapid dissociation of the bisdemethoxycurcumin moiety from complex **4a** greatly enhanced its activity and selectivity against targeted cancer cells compared with the parent curcumin-containing complex.

### 4.2. Amino Acids

**Figure 6:**
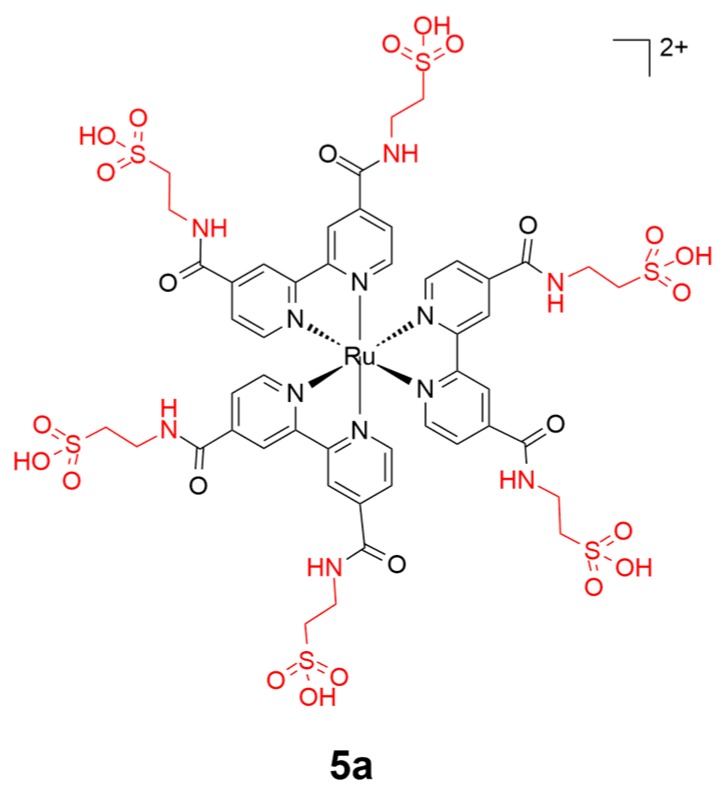
Chemical structure of complex **5a**. The taurine motif is highlighted in red. Reprinted figure with permission from Copyright (2017) Royal Society of Chemistry.

Taurine is a typical amino acid existing in the brain and is involved in central nervous system (CNS) signaling. A taurine-bearing ruthenium(II) compound (**5a**) has been reported by Zhang and coworkers for targeting brain cancer cells (Figure 6) [64]. Complex **5a** showed lysosome-specific intracellular accumulation in cancer cells. The symmetrical introduction of taurine moieties to the ruthenium(II) scaffold benefits the enhancement of the emission and further releases of ROS, which renders the taurine-conjugated molecule a reactive photosensitizer for photodynamic therapy to target tumor cells selectively.

**Figure 7:**
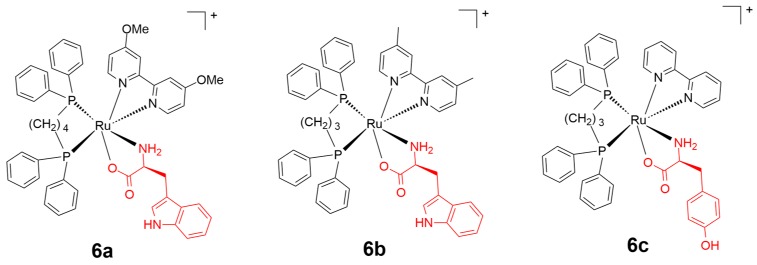
Chemical structure of complexes **6a**, **6b**, and **6c**. The amino acid residues are highlighted in red. Reprinted figure with permission from Copyright (2017) Elsevier Inc. and Copyright (2017) Elsevier B.V.

Ruthenium(II) complexes bearing proteinogenic α-amino acids exhibit potentially reduced cytotoxicity over cancer cells in contrast to anticancer platinum complexes [64]. In 2018, Santos and coworkers [65] developed thirteen amino acid conjugated ruthenium(II) complexes as cancer therapeutic drugs, including l-methionine (Met), l-histidine (His), l-tryptophan (Trp), l-tyrosine (Tyr), l-valine (Val), l-alanine (Ala), and glycine (Gly). Human breast cancer cells and healthy breast cells (MDA-MB-231 and MCF-10A, respectively) were applied for in vitro cytotoxicity investigation, and cisplatin was used as the reference drug. All of the scaffolds exhibited inhibition activity and selectivity to MDA-MB-231 over MCF-10A with promising IC_50_ values contrasted to the reference drug cisplatin. Complexes **6a** and **6b** containing l-Trp residue showed the best combination of activity and selectivity (Figure 7). Particularly, complex **6b** (IC_50_ = 3.0 and 29.9 µM, respectively) showed better anticancer selectivity to cancer cells over normal cell lines, and a higher anticancer efficiency over non-amino acid conjugated ruthenium(II) complexes (IC_50_ = 15.6 and 17.0 µM, respectively). Moreover, this group developed similar amino acid-based ruthenium(II) complexes and evaluated their activity against MDA-MB-231 cells. These conjugates demonstrated a better activity against breast cancer cells compared to the reference drug cisplatin and the non-amino acid conjugate ruthenium(II) complex, with an IC_50_ ranging from 3.04 to 7.44 µM. Among all of the developed compounds, complex **5c** bearing the Tyr residue exhibited the best activity [66].

### 4.3. Lipids

**Figure 8:**
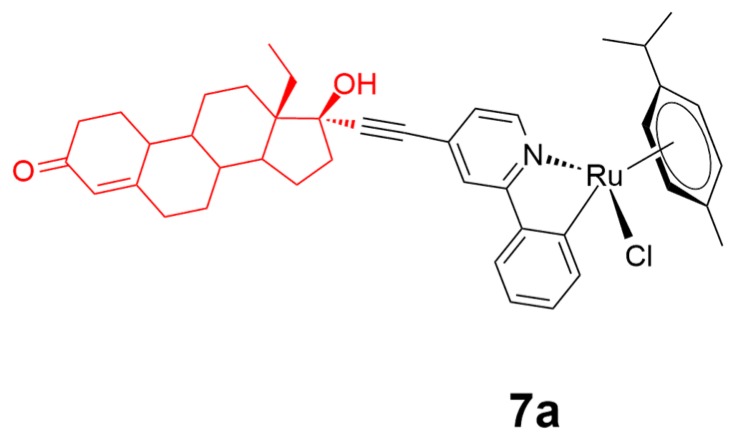
Chemical structure of complex **7a**. The levonorgestrel group is highlighted in red. Reprinted figure with permission from Copyright (2011) American Chemical Society.

The binding affinity towards targeted active sites is of great importance for cancer therapy development based on the similar hydrophilic–lipophilic ability of the treatment molecule and the corresponding targeting receptors [66]. Ruiz and coworkers recently developed a lipophilic levonorgestrel-conjugated ruthenium(II) complex **7a** (Figure 8) against breast cancer [67]. Complex **7a** showed an enhanced inhibition activity against T47D breast cancer cells over nonsteroidal analogues with an eight-fold activity enhancement over the reference cisplatin. The conjugation of the levonorgestrel motif to ruthenium(II) scaffold generated a tunable synergy between the steroidal axial-accessories and the ruthenium(II) metal center, thus benefiting the improved activity of the comprehensive scaffold. Theoretical density functional theory calculations on complex **7a** suggested that the lipophilic steroidal moiety increased the lability of the Ru–Cl bond, allowing for the easier formation of a stronger Ru–N bond upon substitution of Cl by N-nucleophiles. The calculations also revealed the interaction of the guanine/phenylpyridine/steroid moiety towards the reception targets at the lowest energy located in pseudocavity.

**Figure 9:**
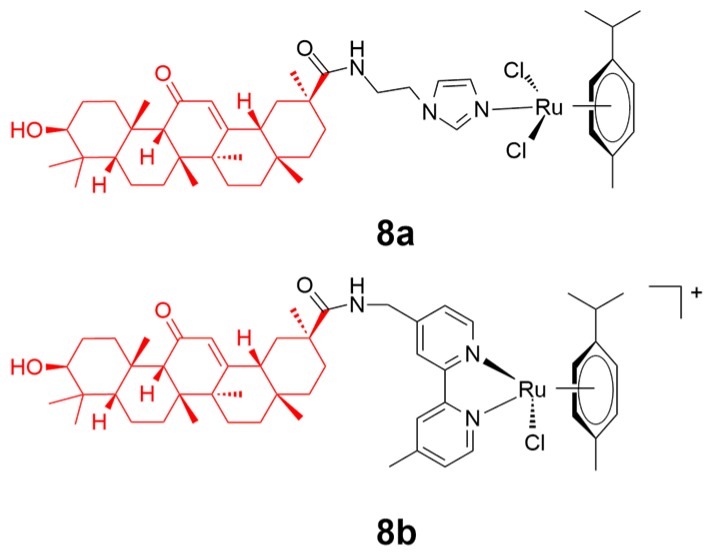
Chemical structure of complexes **8a** and **8b**. The glycyrrhetinic acid motif is highlighted in red. Reprinted figure with permission from Copyright (2018) Elsevier Inc.

Glycyrrhetinic acid (GA) widely exists in Glycyrrhiza glabra and possesses outstanding anticancer activity through multiple mechanisms, including ROS production [68], mitochondria targeting [68], acting via protein receptors [68], or affecting the microenvironment of the tumor cell [69]. Liu and coworkers reported the conjugation of 18β-glycyrrhetinic acid on ruthenium(II)-arene scaffolds to generate conjugates **8a** and **8b** (Figure 9) [70]. Complex **8b** containing the *N*,*N*-chelating moiety rather than the imidazole moiety in **8a** displayed a higher stability and better solubility. Complex **8b** experienced a higher rate of hydrolysis than **8a**, with only one chloride leaving motif within the scaffold. Additionally, complex **8b** also exhibited a better inhibition towards human cancer cells. This might be attributed to the primary role that complex **8b** participated in the alteration of secondary structure of B-DNA by hydrolysis, and in the enhancement of intracellular ROS concentrations, which is likely to cause the disruption of cell metabolism and/or cell death.

### 4.4. Carbohydrates

**Figure 10:**
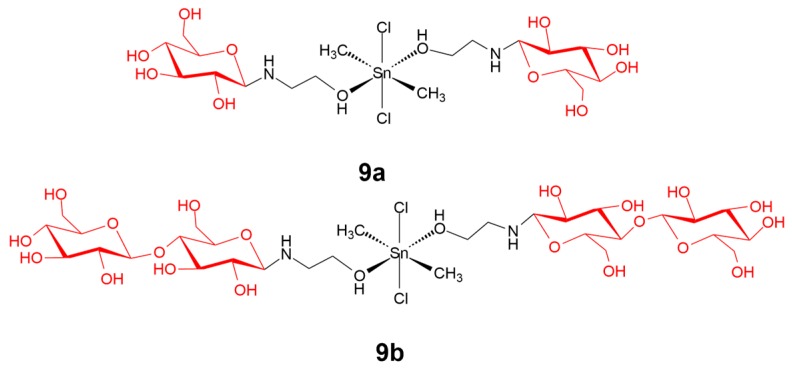
Chemical structure of complexes **9a** and **9b**. The carbohydrate moieties are highlighted in red. Reprinted figure with permission from Copyright (2013) Royal Society of Chemistry.

Carbohydrates are widely distributed natural compounds and possess multiple tunable functional groups for modulation according to their desired properties. In living systems, carbohydrates play an important role in mediating carbohydrate–protein interactions, which are crucial in cell–cell recognition and adhesion phenomena during cancer growth and progression [71]. Tabassum and coworkers [72] recently described carbohydrate-linked organotin(IV) complex **9a** and **9b**, as a human topoisomerase Iα inhibitor against human carcinoma cells (Figure 10). Both of the complexes showed a strong Topo I inhibition activity in contrast to the reference drug cisplatin at 30–35 µM. The complexes also showed good antiproliferative activity against human carcinoma cells. The complexes significantly suppressed the expression of MMP-2 mRNA levels, suggesting that their antiproliferative activity was mediated through inducing morphological transformations and further cell apoptosis.

### 4.5. Vitamin

**Figure 11:**
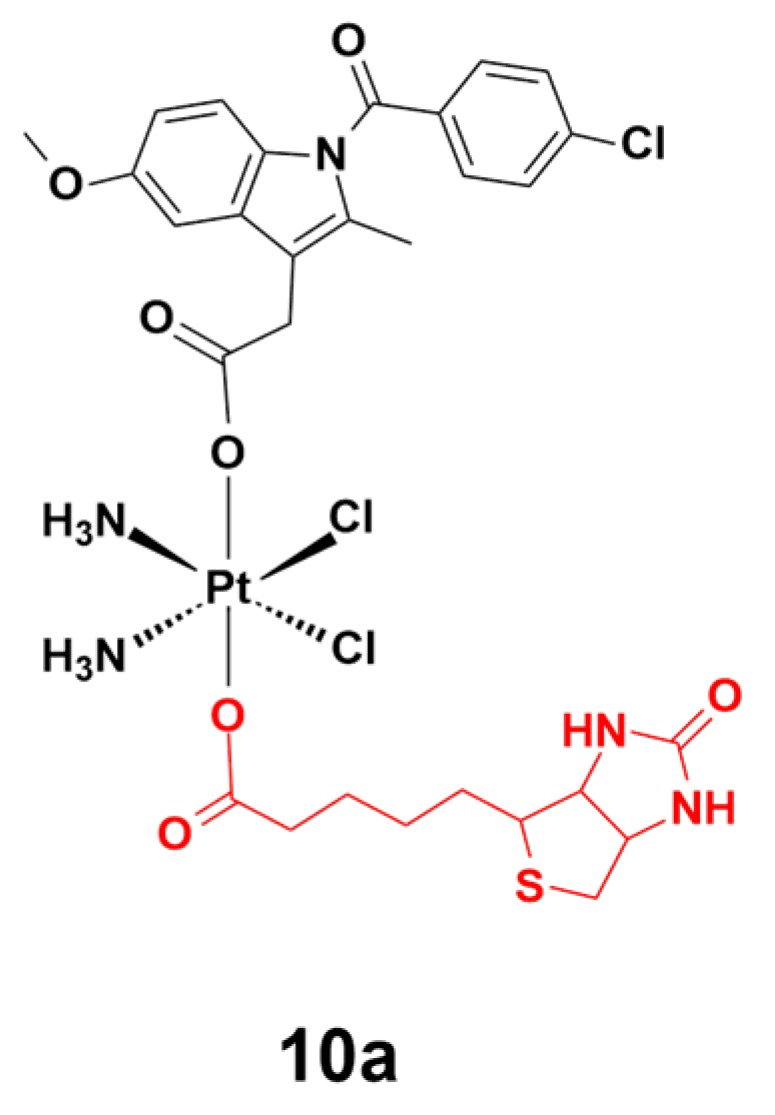
Chemical structure of complex **10a**. The biotin moiety is highlighted in red. Reprinted figure with permission from Copyright (2017) Elsevier Inc.

Biotin is a water-soluble vitamin that is involved in the metabolism of amino acids, carbohydrates, and fats, both in humans and in other organisms. Gou and coworkers recently reported biotin-conjugated platinum(IV) complex **10a**, which selectively targets cancer cells expressing enhanced levels of biotin receptor (Figure 11) [73]. Complex **10a** was activated by endogenous reductants in the cellular environment, to release indomethacin and cisplatin moieties to inhibit cancer cell activity. In in vitro studies, complex **10a** displayed a remarkable activity against cisplatin-resistant gastric cancer cells (SGC7901/CDDP), as well as five other cancer cell lines, including gastric cancer cells (SGC7901), umbilical vein endothelial cells (EA. hy926), prostate carcinoma cells (PC-3), hepatocellular carcinoma cells (HepG-2), and colorectal cancer cells (HCT-116). Notably, complex **10a** also alleviated inflammatory symptoms in cancer cells via the inhibition of cyclooxygenases. Moreover, complex **10a** perturbed the formation of capillary-like tubes in EA. hy 926 cells and weakened the invasiveness of the highly aggressive PC-3 cell line.

## 5. Conclusions

Most cancer-oriented metal therapies attract significant interest in the activity levels against cancer cells/tissues. Focus on the toxicity and selectivity over healthy surroundings, including cells, tissues, or organs, is somehow neglected and rendered as secondary factors for consideration, which is, however, the primary concern for patients suffering from severe cancer sickness. In 2016, about nine million people worldwide were reported to have died from diverse species of cancers. Hence, growing efforts have been contributed to give a closer look and a more comprehensive understanding of the function mechanisms as well as the corresponding side effects from cancer therapies, particularly for metal-conjugated complexes bearing inherent toxicity from the metals. In search of side-effect reduced metal therapies, tremendous improvements in terms of the sensitivity, efficiency, selectivity, and stability have been achieved, capitalizing on the unique characteristics of metal centers, including distinguished spectroscopic properties and isotopic patterns.

Side effects from metal-centered cancer therapies often differ from species to species, owing to their diversity of the conjugated ligands and mechanisms of action. To date, platinum drugs are still the most important class of metal-based compounds used in the clinic, however, resistance and adverse side effects are limiting factors against their more widespread use. In this context, strategies for the modification of alternative metal centers (such as ruthenium(II), iridium(III), osmium(IV), gold(III), palladium(II), rhodium(III), iron(III), cobalt(II), and tin(II)) or axial conjugating ligands (either based on synthesized organic moiety or natural product-based motif) continue to be sought in order to further the development of novel metal-based drugs with tunable medicinal characteristics, improved potency, and reduced side effects. Particularly, natural products possess inherent benefits with abundant structure diversity; effortless access; and, most importantly, splendid bioactivity with excellent biocompatibility, mainly based on hydrolysis and redox processes upon binding with intercellular/intracellular target sites. Except for small molecule-based natural product moieties, such as naturally derived active ingredients, amino acids, lipids, carbohydrates, or vitamins, larger natural biomolecules, including peptides [74,75,76], antibodies [77,78] and oligonucleotides, particularly aptamers [79,80,81], have also been introduced into metal-based complexes as potential cancer therapies. Linking biomolecules to metal complexes can both reduce their cytotoxicity and enhance the bioavailability of the conjugates to the specific target organelles/cells/tissues, owing to their similar physicochemical properties and steric configurations to the target biomolecules. However, as larger biomolecules are extremely sensitive to the surrounding environment, the maintenance of their original biological structure and function upon grafting to a metal scaffold can be rather difficult compared with small molecule-based metal complexes. Looking forward, we speculate that more efforts by pioneers will be contributed to the improvement of function efficiency and selectivity over targeted cancer cells/tissues, based on natural product-conjugated metal complexes. In this case, unwanted side effects can be very likely to be reduced with enhanced cancer targeting and anticancer efficiency.

## Figures and Tables

**Table 1:** A summary table of the latest assays for the development of natural product-conjugated metal complexes as cancer therapies.

Reference	Natural Moiety	Metal Center	Mechanism of Action or Target	Cytotoxicity against Target Cells (IC50)	Cytotoxicity against Normal Cells (IC50)	Reference Compound	Demonstrated Application in
Kang et al., 2017 [51]	Benzofuran	Iridium(III)	Transcription factors NF-κB and STAT3	4.34 μM	29.21 μM (LO2 cells); 32.24 μM (HEK293 cells)	Cisplatin and doxorubicin	Prostate cancer cells (DU145)
Oliveira et al., 2017 [53]	Lapachol	Ruthenium(II)	Bovine serum albumin (BSA) and human serum albumin (HSA)	0.086 μM; 0.09 μM	0.72 μM (V79 cells)	Cisplatin	Breast cancer cells (MDA-MB-231); lung cancer cells (A549)
Beauperin et al., 2017 [56]	Podophyllotoxin	Iron(III)	Reactive oxygen species (ROS)	0.93 μM; 0.43 μM	NA	Podophyllotoxin	Breast cancer cells (MCF-7 and MDA-MB-231)
Pettinari et al., 2014 [63]	Curcumin	Ruthenium(II)	Hydrolysis	0.20 μM; 0.27 μM	13.0 μM (HEK293 cells)	Cisplatin	Ovarian carcinoma cells (A2780 and A2780R)
Du et al., 2017 [64]	Taurine	Ruthenium(II)	Reactive oxygen species (ROS)	NA	NA	Cisplatin and non-natural product conjugates	Brain cancer cells (F98, A375, HeLa, and A549)
Santos et al., 2018 [65]	l-tryptophan (Trp)	Ruthenium(II)	Human serum albumin (HSA)	3.0 μM	29.9 μM (MCF-10A cells)	Cisplatin and non-natural product conjugates	Breast cancer cells (MDA-MB-231)
Santos et al., 2017 [66]	l-tyrosine (Tyr)	Ruthenium(II)	N/A	3.04 μM	NA	Cisplatin	Breast cancer cells (MDA-MB-231)
Ruiz et al., 2011 [67]	Levonorgestrel	Ruthenium(II)	DNA	7.4 μM; 3.7 μM	NA	Cisplatin	Breast cancer cells (T47D); ovarian cancer cells (A2780)
Kong et al., 2018 [70]	Glycyrrhetinic acid	Ruthenium(II)	DNA and ROS	24.2 μM; 34.6 μM; 63.7 μM	NA	Cisplatin	Cervical cancer cells (HeLa)o breast cancer cells (MCF-7); ovarian cancer cells (A278)
Khan et al., 2014 [72]	Carbohydrates	Organotin(IV)	Human topoisomerase Iα	30 μM	NA	NA	Hepatoma cancer cells (Huh7)
Hu et al., 2017 [73]	Vitamin	Platinum(IV)	Endogenous reducing molecules	42.73 μM	59.64 μM (LO-2 cells)	Cisplatin	Umbilical vein endothelial cell (EA. hy926)

## Abbreviations

BSA	bovine serum albumin
HSA	human serum albumin
PPT	podophyllotoxin
ROS	reactive oxygen species
CNS	central nervous system
GA	glycyrrhetinic acid
